# *hapE* and *hmg1* Mutations Are Drivers of *cyp51A*-Independent Pan-Triazole Resistance in an Aspergillus fumigatus Clinical Isolate

**DOI:** 10.1128/spectrum.05188-22

**Published:** 2023-05-04

**Authors:** Ana C. O. Souza, Wenbo Ge, Nathan P. Wiederhold, Jeffrey M. Rybak, Jarrod R. Fortwendel, P. David Rogers

**Affiliations:** a Department of Pharmacy and Pharmaceutical Sciences, St. Jude Children’s Research Hospital, Memphis, Tennessee, USA; b Department of Clinical Pharmacy and Translational Sciences, University of Tennessee Health Science Center, Memphis, Tennessee, USA; c Fungus Testing Laboratory, Department of Pathology and Laboratory Medicine, University of Texas Health Science Center at San Antonio, San Antonio, Texas, USA; University of Michigan

**Keywords:** *Aspergillus fumigatus*, triazole drug resistance, *hmg1*, *hapE*, CCAAT box binding complex

## Abstract

Aspergillus fumigatus is a ubiquitous environmental mold that can cause severe disease in immunocompromised patients and chronic disease in individuals with underlying lung conditions. Triazoles are the most widely used class of antifungal drugs to treat A. fumigatus infections, but their use in the clinic is threatened by the emergence of triazole-resistant isolates worldwide, reinforcing the need for a better understanding of resistance mechanisms. The predominant mechanisms of A. fumigatus triazole resistance involve mutations affecting the promoter region or coding sequence of the target enzyme of the triazoles, Cyp51A. However, triazole-resistant isolates without *cyp51A*-associated mutations are frequently identified. In this study, we investigate a pan-triazole-resistant clinical isolate, DI15-105, that simultaneously carries the mutations *hapE*^P88L^ and *hmg1*^F262del^, with no mutations in *cyp51A*. Using a Cas9-mediated gene-editing system, *hapE*^P88L^ and *hmg1*^F262del^ mutations were reverted in DI15-105. Here, we show that the combination of these mutations accounts for pan-triazole resistance in DI15-105. To our knowledge, DI15-105 is the first clinical isolate reported to simultaneously carry mutations in *hapE* and *hmg1* and only the second with the *hapE*^P88L^ mutation.

**IMPORTANCE** Triazole resistance is an important cause of treatment failure and high mortality rates for A. fumigatus human infections. Although Cyp51A-associated mutations are frequently identified as the cause of A. fumigatus triazole resistance, they do not explain the resistance phenotypes for several isolates. In this study, we demonstrate that *hapE* and *hmg1* mutations additively contribute to pan-triazole resistance in an A. fumigatus clinical isolate lacking *cyp51*-associated mutations. Our results exemplify the importance of and the need for a better understanding of *cyp51A*-independent triazole resistance mechanisms.

## INTRODUCTION

Aspergillus fumigatus is a ubiquitous environmental mold, spores of which are commonly inhaled by humans (1). While innocuous in immunocompetent individuals, A. fumigatus can cause life-threatening disease when the host immune system is compromised, especially in patients undergoing hematopoietic stem cell or solid organ transplantation (2–5). A. fumigatus infections can also manifest as chronic disease in individuals with certain underlying lung conditions, such as asthma, lung cavitary disease (e.g., tuberculosis or sarcoidosis), cystic fibrosis, or chronic obstructive pulmonary disease (6–8). It is estimated that over 200,000 life-threatening Aspergillus infections occur every year, with mortality rates as high as 20 to 90% (9–12).

Mold-active triazoles are first-line therapy against A. fumigatus infections (13). Triazole antifungals inhibit the A. fumigatus sterol demethylases Cyp51A and Cyp51B (14), causing an impairment in ergosterol biosynthesis and an accumulation of toxic sterols that culminates in fungicidal activity (15–18). Currently, four triazole drugs have utility for the management of Aspergillus infections, i.e., voriconazole (VRC), itraconazole (ITRA), posaconazole (POS), and isavuconazole (ISA) (13). Unfortunately, an alarming number of triazole-resistant clinical and environmental A. fumigatus isolates have been reported worldwide, encouraging the search for novel therapeutic strategies and reinforcing the need for a better understanding of antifungal resistance mechanisms (19, 20).

The predominant mechanism of triazole resistance in A. fumigatus is genetic mutation leading to changes in the amino acid sequence and/or expression levels of one of the two target enzymes, Cyp51A (19, 20). Tandem repeats (TRs) in the promoter of the *cyp51A* gene coupled with single point mutations in the coding sequence, such as TR_34_/L98H or TR_46_/Y121F/T289A, have been widely reported in clinical and environmental isolates. Such TRs in the promoter lead to upregulation of *cyp51A* expression, which in turn increases the amount of triazole necessary for enzyme inhibition (21, 22). Moreover, amino acid substitutions in hot spot residues, such as G54 and M220, as well as those occurring with TRs in the promoter, are thought to cause conformational changes that reduce the binding affinity of the triazole drugs with the target enzyme (19, 20, 23, 24).

While *cyp51A*-associated mutations are the most common and best-characterized mechanism of triazole drug resistance, non-*cyp51A* triazole-resistant clinical isolates have been increasingly reported, with prevalence rates varying from 15 to 60% depending on geographic region (25–28). However, *cyp51A*-indepent triazole drug resistance remains poorly understood and underinvestigated. One of the potential causes of triazole drug resistance is the overexpression of ATP binding cassette (ABC) and major facilitator superfamily (MFS) multidrug transporters (29–31), which is often observed among triazole-resistant A. fumigatus isolates. Overexpression of these transporters is thought to cause a decrease in intracellular drug concentrations, therefore decreasing the amount of drug available to inhibit sterol demethylases (29–31).

Mutations in *hmg1*, which encodes the 3-hydroxy-3-methylglutaryl-coenzyme A (HMG-CoA) reductase Hmg1, have also recently emerged as a genetic determinant of triazole drug resistance in A. fumigatus (26). Hmg1 initiates ergosterol biosynthesis by catalyzing the reduction of HMG-CoA into mevalonate and has a conserved motif known as the sterol-sensing domain (SSD), which is predicted to participate in the regulation of HMG-CoA reductase activity (32–34). Although the clinical prevalence of *hmg1* mutations has not been assessed in a systematic manner, such mutations have been identified in over 150 isolates thus far reported in the literature, many of which are resistant to triazole drugs and lack *cyp51A*-associated mutations (25–28, 35–44). The most frequent Hmg1 amino acid substitutions associated with triazole drug resistance occur in residues putatively located in the Hmg1 SSD, such as S269, S305, G307, and I412, with several studies showing that some of these mutations, namely, F262del, S305P, I412S, S269F, and E306K, impart decreased susceptibility to multiple triazoles when genetically introduced into a susceptible laboratory strain (25, 26, 36, 40). The mechanism by which *hmg1* mutations alter triazole resistance remains to be defined; however, it is speculated that amino acid substitutions in the SSD impair its ability to sense sterols and signal for Hmg1 degradation (25, 26, 28).

Another recently identified mechanism of triazole drug resistance involves mutations in the *hapE* gene, which encodes one of the three subunits of the CCAAT box binding complex (CBC), a transcription factor that has been shown to repress the expression of several ergosterol biosynthesis genes, including *cyp51A* (45, 46). It has been suggested that the *hapE*^P88L^ mutation decreases the binding affinity of the CBC for the promoter region of *cyp51A* (47), impairing its ability to repress gene expression, which culminates in overexpression of *cyp51A*. Thus far, *hapE* mutations seem to be rare occurrences, having been reported for only 3 isolates (26, 46, 48).

In this study, we investigated a pan-triazole-resistant clinical isolate, DI15-105, that simultaneously carries the mutations *hapE*^P88L^ and *hmg1*^F262del^ and lacks mutations in *cyp51A*. Our goal was to determine the impact of these mutations individually and in combination on the triazole resistance phenotype of DI15-105. Using a Cas9-mediated gene-editing system, *hapE*^P88L^ and *hmg1*^F262del^ mutations were reverted in DI15-105. Our results demonstrated that, together, these mutations account for the high levels of pan-triazole resistance in DI15-105.

## RESULTS

### Correction of the *hapE*^P88L^ and *hmg1*^F262del^ mutations recovers DI15-105 susceptibility to triazole drugs.

**FIG 1:**
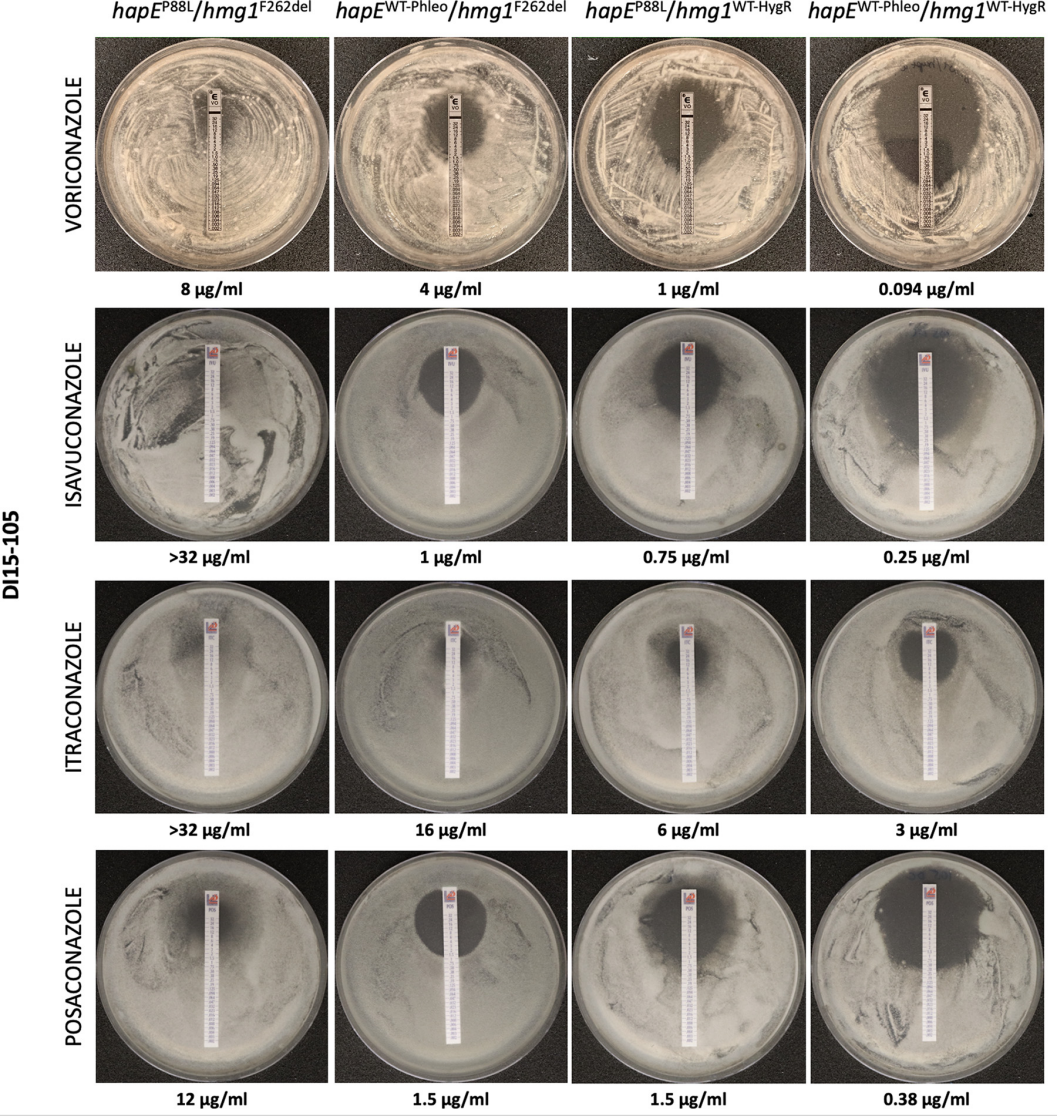
Antifungal susceptibility of A. fumigatus DI15-105 clinical isolates after correction of *hapE*^P88L^ and *hmg1*^F262del^ mutations, using gradient diffusion test strips. Conidia were inoculated in RPMI 1640 agar with VRC, ISA, ITRA, or POS gradient diffusion test strips and incubated at 35°C for 48 h. MICs were determined as the concentration at which a zone of growth clearance intercepted the drug strip; MIC values are indicated at the bottom of each image.

The *hapE*^P88L^ and *hmg1*^F262del^ mutations have been individually observed in triazole-resistant clinical isolates and independently shown to influence triazole susceptibility (25, 26, 46). Using *in vitro*-assembled Cas9 ribonucleoproteins (RNPs), allele swaps were performed and successfully replaced the mutated versions of *hapE* and *hmg1* (previously performed [26]) with their respective wild-type (WT) versions (derived from AF293) in the clinical isolate DI15-105, as confirmed by PCR screening (see Fig. S1 and S2 in the supplemental material) and Sanger sequencing (data not shown). Using gradient diffusion test strips (Fig. 1), triazole susceptibility was determined and revealed that correction of either *hapE* or *hmg1* gene sequences decreased DI15-105 MICs by 2-fold and 5-fold, respectively. MIC values decreased even further, ≥10-fold, when *hapE* and *hmg1* gene sequences were both corrected.

**FIG 2:**
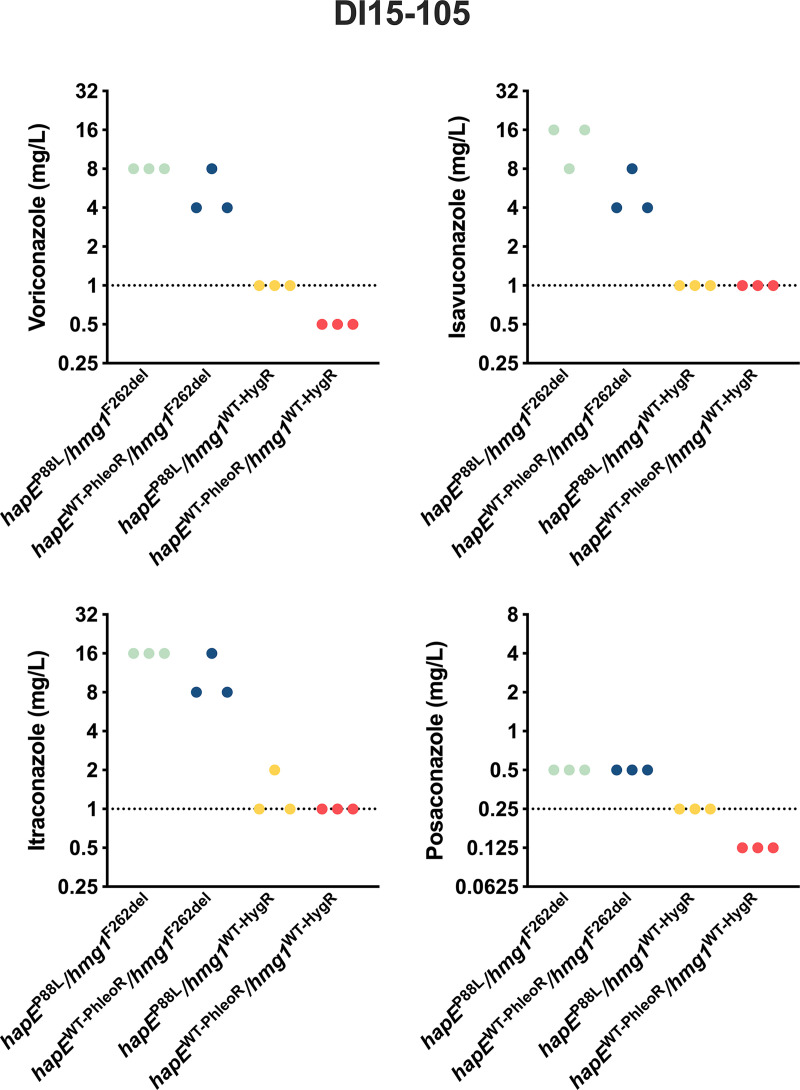
Antifungal susceptibility of A. fumigatus DI15-105 clinical isolates after correction of *hapE*^P88L^ and *hmg1*^F262del^ mutations, using triazole broth microdilution assays. Triazole MICs were determined according to CLSI methodology. VRC, ISA, ITRA, and POS MICs of triplicates are represented, and dotted lines indicate CLSI epidemiological cutoff values for each triazole drug.

Triazole susceptibility of DI15-105 and derived strains was also assessed by broth microdilution assays according to the Clinical and Laboratory Standards Institute (CLSI) methodology (49) (Fig. 2). Correction of the *hapE* mutation in DI15-105_*hapE*^WT-PhleoR^/*hmg1*^F262del^ decreased the MICs for VRC, ISA, and ITRA by 2-fold, while correction of the *hmg1* mutation in DI15-105_*hapE*^P88L^/*hmg1*^WT-HygR^ decreased the MICs for all triazoles (VRC and ISA, 8-fold; ITRA, 16-fold; POS, 2-fold) (Fig. 2). Correction of both *hapE* and *hmg1* mutations in DI15-105_*hapE*^WT-PhleoR^/*hmg1*^WT-HygR^ fully restored triazole susceptibility of DI15-105 as established by the CLSI epidemiological cutoff values, reducing the MIC levels by 4-fold (POS) to 16-fold (VRC, ISA, and ITRA).

### Correction of the *hapE*^P88L^ and *hmg1*^F262del^ mutations in DI15-105 reduces *cyp51A* expression levels.

**FIG 3:**
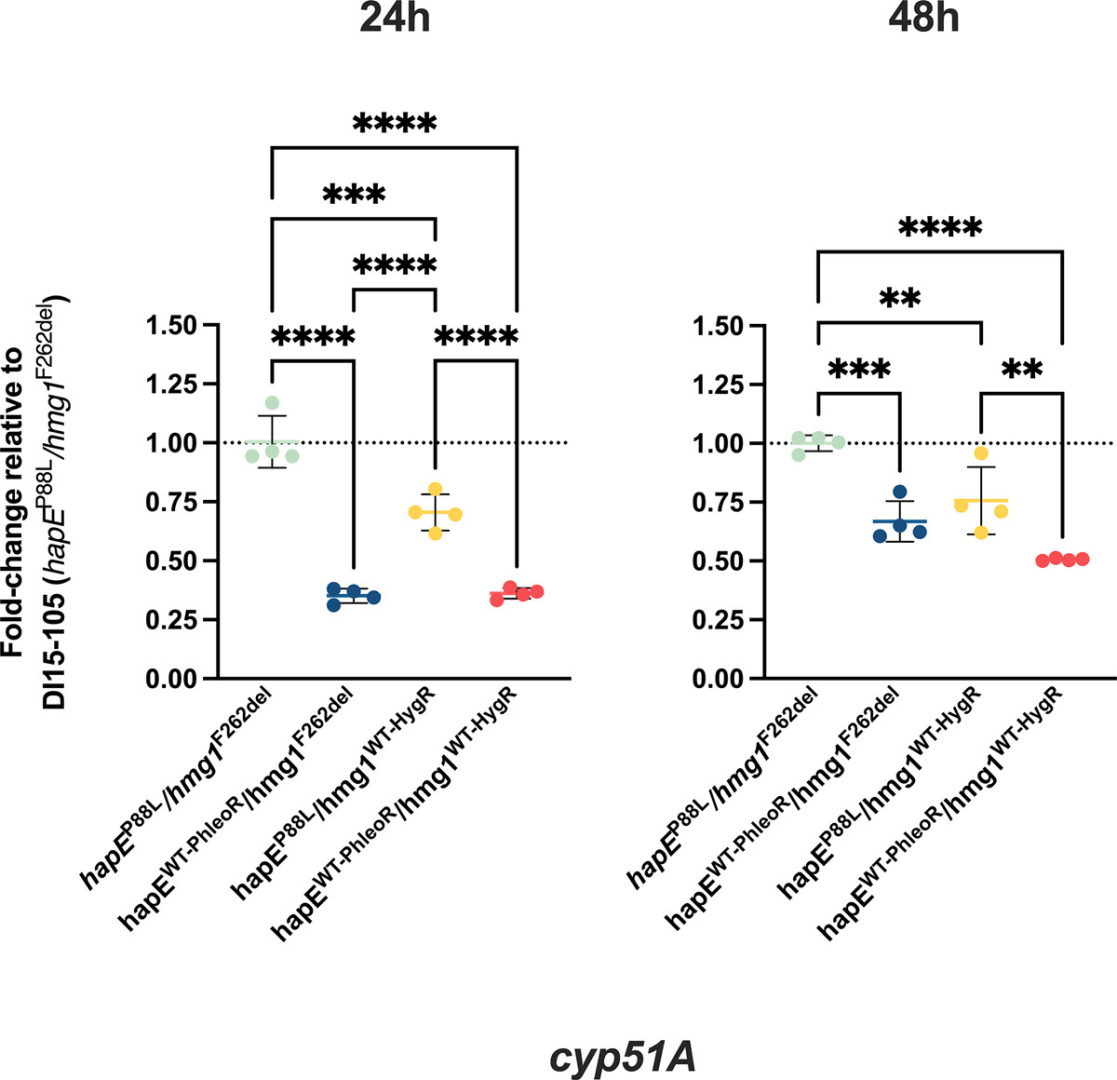
Expression levels of *cyp51A* in DI15-105 isolates after correction of *hapE*^P88L^ and *hmg1*^F262del^ mutations. The *cyp51A* expression levels of *hmg1* and *hapE* correction strains were assessed using RT-qPCR. RPMI 1640 medium was inoculated with A. fumigatus conidia at a concentration of 2 × 10^4^ conidia/mL and incubated at 35°C and 250 rpm for 24 h or 48 h. Mycelia were harvested and subjected to RNA extraction. cDNA was synthesized, and qPCR was performed using *tubA*, which encodes β-tubulin A, as the housekeeping gene for data normalization. Changes in gene expression among isolates were calculated using the 2^−ΔΔ^*^CT^* method. Experiments were performed using four biological replicates. Statistical analysis was performed using one-way ANOVA with Tukey’s multiple-comparison test. **, *P* < 0.01; ***, *P* < 0.001; ****, *P* < 0.0001.

It has been suggested that the *hapE*^P88L^ mutation reduces the affinity of CBC for the promoter region of *cyp51A*, thus impairing its ability to repress gene expression. This ultimately leads to an increase in *cyp51A* expression, resulting in increased MICs for triazole drugs. It is not clear by what mechanism *hmg1* mutations, particularly *hmg1*^F262del^, increase triazole resistance, and previous studies have not reported changes in *cyp51A* expression. Since we observed an additive effect of these two mutations contributing to triazole resistance, we assessed expression levels of *cyp51A* upon correction of *hapE*^P88L^ and *hmg1*^F262del^ mutations. As expected, correction of the *hapE* mutation in both DI15-105_*hapE*^WT-PhleoR^/*hmg1*^F262del^ and DI15-105_*hapE*^WT-PhleoR^/*hmg1*^WT-HygR^ significantly decreased expression of *cyp51A* after 24 h (~2.8-fold and ~2.7-fold, respectively) and 48 h (~1.5-fold and ~2-fold, respectively) of fungal growth (Fig. 3). Correction of *hmg1* also caused a less prominent but statistically significant decrease in *cyp51A* expression at 24 h (~1.4-fold) and 48 h (~1.3-fold) of fungal growth.

### Correction of the *hapE*^P88L^ mutation improves DI15-105 radial growth and induces hypersensitivity to oxidative stress.

**FIG 4:**
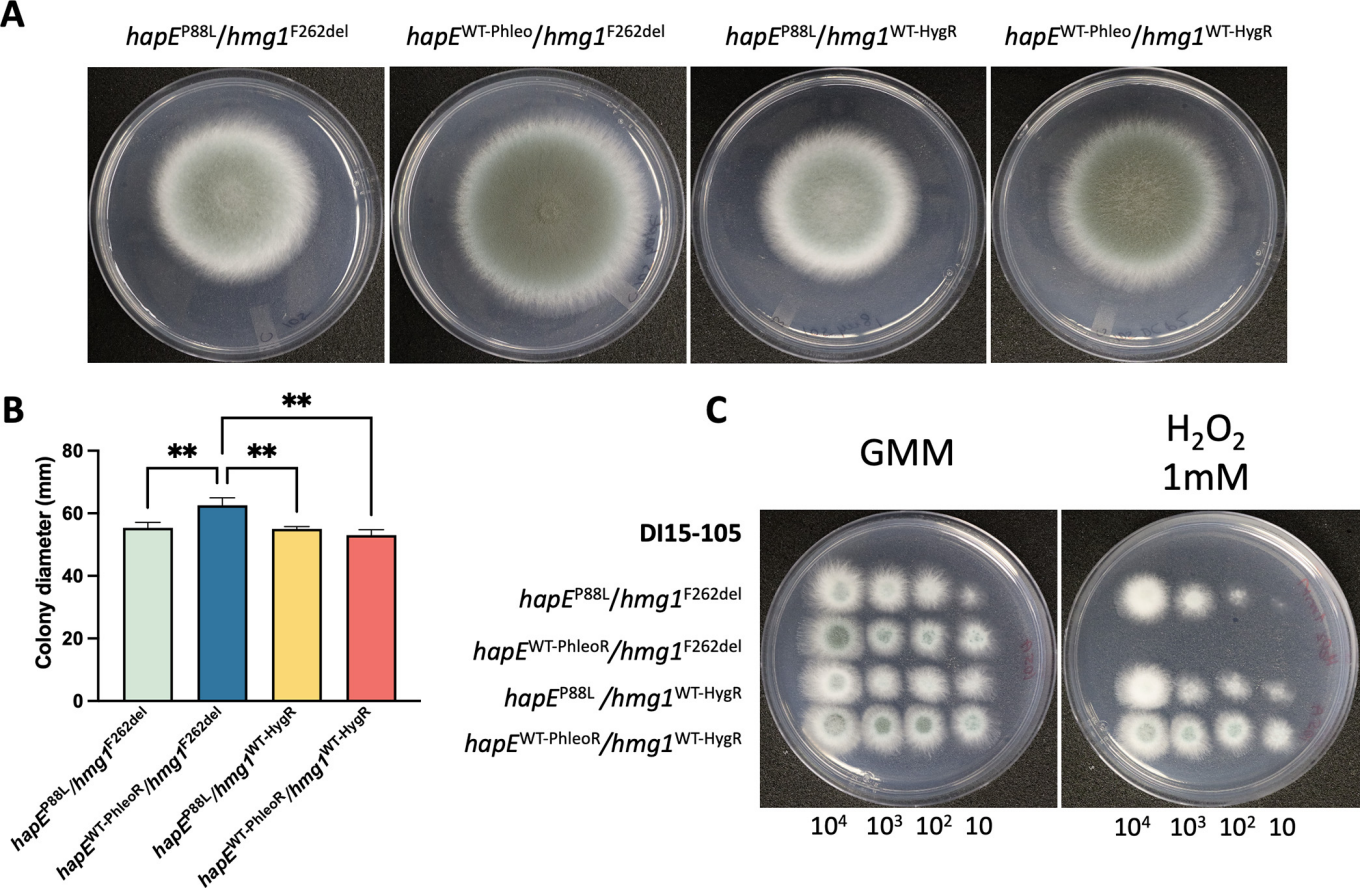
Phenotypic characterization of DI15-105 isolates after correction of *hapE*^P88L^ and *hmg1*^F262del^ mutations. (A and B) Macromorphology (A) and radial growth (B) were assessed after point inoculation of 10^4^ A. fumigatus conidia/5 μL in GMM agar and incubation for 96 h at 37°C. (C) Fungal growth under oxidative stress was analyzed through spot dilution assays in GMM agar supplemented with 2 mM H_2_O_2_ using 5-μL suspensions containing 10^4^, 10^3,^ 10^2^, or 10 conidia/5 μL, followed by incubation at 37°C for 48 h. Experiments were performed using three biological replicates. Statistical analysis was performed using one-way ANOVA with Tukey’s multiple-comparison test. **, *P* < 0.01.

In some studies, the *hapE*^P88L^ mutation has been associated with decreased fungal growth (45, 47). Therefore, we analyzed colony macromorphology (Fig. 4A) and radial growth (Fig. 4B) of the DI15-105 isolate and derived strains. Macromorphological analysis revealed that the strain with a *hapE* single correction (DI15-105_*hapE*^WT-PhleoR^/*hmg1*^F262del^) presented significantly enhanced radial growth, which was not observed in the double correction strain DI15-105_*hapE*^WT-PhleoR^/*hmg1*^WT-HygR^. We also observed that the *hapE* correction strains DI15-105_*hapE*^WT-PhleoR^/*hmg1*^F262del^ and DI15-105_*hapE*^WT-PhleoR^/*hmg1*^WT-HygR^ presented a darker green color than *hapE*^P88L^ strains, suggesting greater conidium production; however, quantification of conidia did not show significant differences in the amounts of conidia per colony area among the isolates (Fig. S3).

In order to evaluate the impact of *hmg1* and *hapE* mutations on the DI15-105 stress response, strains were grown in agar medium supplemented with several stressors, namely, H_2_O_2_ (oxidative stress) (Fig. 4C), iron (depletion and excess amounts) (see Fig. S4A), calcofluor white and Congo red (cell wall stress) (see Fig. S4B), and sorbitol (osmotic stress) (see Fig. S4B). Only correction of the *hapE*^P88L^ mutation in DI15-105_*hapE*^WT-PhleoR^/*hmg1*^F262del^ rendered the isolate hypersensitive to oxidative stress, which once again was not observed when the *hmg1* mutation was also corrected (Fig. 4; also see Fig. S5).

## DISCUSSION

The emergence of triazole drug resistance represents an important challenge in the therapy of A. fumigatus infections (50). Genetic alterations in the *cyp51A* gene coding sequence or promoter region are the most frequently identified and best-characterized cause of A. fumigatus clinical triazole resistance. However, resistance to triazoles is not always explained by genetic alterations in *cyp51A* (25, 26, 28, 35, 46, 48, 51). In an earlier study, whole-genome sequencing of A. fumigatus clinical isolates collected in the United States revealed a clinical isolate, DI15-105, that had high levels of resistance to mold-active triazoles and no mutations in *cyp51A* (26). Instead, this isolate was shown to carry the *hmg1*^F262del^ and *hapE*^P88L^ mutations. Both mutations were previously reported separately in A. fumigatus clinical isolates and shown to directly contribute to triazole drug resistance (26, 46). Here, our goal was to determine the impact of these mutations individually and in combination on the triazole resistance phenotype of DI15-105.

The *hmg1*^F262del^ mutation is predicted to alter a residue in the SSD of Hmg1, a conserved motif shown to be essential for the negative regulation of sterol biosynthesis in other eukaryotes, including Schizosaccharomyces pombe (32, 33, 52). We previously observed that restoration of the *hmg1* WT sequence in DI15-105 restored triazole susceptibility to all mold-active triazoles (26). The same *hmg1*^F262del^ mutation introduced in a susceptible A. fumigatus laboratory strain induced increases of at least 4-fold in the MICs for all triazoles, accumulation of ergosterol precursors, and significant increases in total cellular ergosterol levels (26). In a similar way, amino acid substitutions in other residues of the Hmg1 SSD have also been identified, and they are often associated with triazole-resistant isolates (25–27, 35, 36). Some of these mutations, namely, S305P, I412S, S269F, and E306K, have been shown to impart decreased susceptibility to multiple triazoles when genetically inserted in a triazole-susceptible laboratory strain (25, 26, 36). It is important to note that many of the *hmg1* mutations so far reported in the literature occur in isolates that also carry *cyp51A* mutations that are known to drive triazole drug resistance (25–28, 36). However, it was demonstrated that, in some cases, the presence of the *hmg1* mutation led to atypical and increased triazole MIC levels in the isolates (26, 27). For instance, genetic reversion of the *cyp51A* mutations TR_34_/L98H, G138C and M263I to the WT sequence did not recover triazole susceptibility in different clinical isolates (26). Those isolates also carried the *hmg1* mutations E105K, Y250H, and I412S, respectively; the latter was previously shown to contribute directly to triazole resistance (26). Additionally, in a clinical isolate carrying the *cyp51A*^M220I^ amino acid substitution, reversion of the *hmg1*^S305P^ mutation reduced the VRC MIC while maintaining ITRA and POS MIC values (36). These data support the evidence that *hmg1* mutations are important determinants of clinical triazole resistance. However, the mechanism by which Hmg1 SSD mutations lead to triazole resistance remains to be fully elucidated, and it is speculated that such mutations might affect the negative regulation of HMG-CoA reductase activity (25, 26).

The *hapE*^P88L^ mutation present in DI15-105 was previously reported in a single clinical isolate that did not also carry mutations in *cyp51A* (46). The *hapE* gene encodes one of the three subunits of the CBC, a highly conserved transcription factor that has been shown to repress expression of several ergosterol biosynthesis genes, such as *cyp51A*, *cyp51B*, *erg7B*, and *erg13B* (45). It has been demonstrated that the *hapE*^P88L^ mutation decreases the binding affinity of the CBC for the promoter region of *cyp51A*, impairing its ability to repress gene expression, which culminates in overexpression of *cyp51A* and thus increased tolerance to triazoles (47). Our results show that the single correction of the *hapE*^P88L^ mutation in DI15-105_*hapE*^WT-PhleoR^/*hmg1*^F262del^ leads to significant decreases in triazole MICs. Recently, a triazole-resistant clinical isolate without *cyp51A* mutations but carrying a HapE splicing site mutation (c.154-1G>A) was identified; it presented higher *cyp51A* expression levels than did an isogenic and triazole-susceptible isolate that had been collected previously from the same patient (48). However, the direct effect of this *hapE* splicing site mutation on triazole resistance has not yet been characterized. Interestingly, in both reports, *hapE* mutations in clinical isolates were thought to have been acquired in the patient after prolonged therapy with triazoles (47, 48). To our knowledge, DI15-105 is only the second isolate that has been reported to carry the *hapE*^P88L^ mutation (26). These examples reinforce the clinical importance of *hapE* mutations for triazole resistance.

In our study, we observed that the *hmg1*^F262del^ mutation has a major impact on the DI15-105 triazole resistance phenotype, since the reversion of this mutation generated a greater decrease in triazole MICs than did the correction of the *hapE*^P88L^ mutation, in both strip and broth microdilution assays. In addition, correction of both *hmg1*^F262del^ and *hapE*^P88L^ mutations in DI15-105_*hapE*^WT-PhleoR^/*hmg1*^WT-HygR^ led to a greater reduction in triazole MICs, demonstrating the additive effects of the two mutations on triazole resistance. In order to elucidate how the two mutations enhanced triazole resistance, we evaluated whether the increased susceptibility of the correction strains to triazole drugs was a consequence of *cyp51A* expression downregulation. Reverse transcription-quantitative PCR (RT-qPCR) results showed a statistically significant but slight decrease in *cyp51A* expression in all correction strains, although the decrease in *cyp51A* expression levels was more prominent in the DI15-105_*hapE*^WT-PhleoR^/*hmg1*^F262del^ and DI15-105_*hapE*^WT-PhleoR^/*hmg1*^WT-HygR^ strains, in which the *hapE* mutation was corrected. Given that the single correction of the *hapE*^P88L^ mutation did not reduce triazole MICs to the extent observed with the reconstitution of both *hapE* and *hmg1* mutations, we think that *cyp51A* expression is not the only mechanism operating in DI15-105. Further investigation is needed to demonstrate how the presence of the *hmg1*^F262del^ and *hapE*^P88L^ mutations together leads to increased MIC values in DI15-105.

In order to identify other ways in which *hmg1*^F262del^ and *hapE*^P88L^ mutations affect A. fumigatus, we analyzed fungal growth and responses to oxidative, iron, osmotic, and cell wall stress in DI15-105 and derived strains. While no significant changes were observed in the DI15-105_*hapE*^P88L^/*hmg1*^WT-HygR^ and DI15-105_*hapE*^WT-PhleoR^/*hmg1*^WT-HygR^ strains, surprisingly a slight improvement in radial growth was detected in the strain in which only the *hapE*^P88L^ mutation was corrected. Although it was not observed in DI15-105_*hapE*^WT-PhleoR^/*hmg1*^WT-HygR^, this recovery of radial growth observed after single correction of *hapE* is consistent with previous reports that showed that the *hapE*^P88L^ mutation, as well as the gene deletion of other subunits of the CBC, generate impairment of fungal growth (45, 47). Moreover, while no unique phenotypes were displayed when strains were grown under iron, osmotic, or cell wall stress conditions, single correction of the *hapE*^P88L^ mutation in DI15-105_*hapE*^WT-PhleoR^/*hmg1*^F262del^ resulted in hypersensitivity to H_2_O_2_. The fact that these phenotypes appear only in the strain in which single correction of *hapE* was performed is intriguing and suggests that, in DI15-105_*hapE*^WT-PhleoR^/*hmg1*^F262del^, recovery of *hapE* ability to regulate gene expression favors fungal growth under basal conditions while also disturbing the strain’s ability to respond to oxidative stress when the *hmg1*^F262del^ mutation is present. Further investigation is required for clarification of this phenomenon.

In summary, we report for the first time that, in a clinical isolate with no *cyp51A*-associated mutations, *hmg1*^F262del^ and *hapE*^P88L^ mutations additively contribute to pan-triazole resistance. Because *cyp51A* expression levels do not seem to entirely explain the phenotypes observed in this study, the mechanism by which the two mutations lead to A. fumigatus triazole resistance remains unclear. This study is another example of the clinical importance of non-*cyp51A*-associated triazole resistance and reinforces the need for a better understanding of the mechanisms that drive triazole resistance in A. fumigatus.

## MATERIALS AND METHODS

### Isolates and growth conditions.

The pan-triazole-resistant clinical isolate DI15-105 was kindly donated by Nathan P. Wiederhold at the Fungus Testing Laboratory at the University of Texas Health Science Center at San Antonio. In a previous study, whole-genome sequencing revealed that this isolate presented no mutations in *cyp51A* but carried the *hapE*^P88L^ and *hmg1*^F262del^ mutations (26). In the same study, a DI15-105-derived strain (DI15-105_ *hapE*^P88L^/*hmg1*^WT-HygR^) in which the *hmg1*^F262del^ mutation was reconstituted with a WT gene sequence was created, and it is also used here.

Fresh conidia from each isolate were obtained after harvesting of 3-day-old glucose minimal medium (GMM) agar cultures at 37°C. The concentrations of conidia in water stocks were then determined using a hemocytometer, and suspensions were kept at 4°C or used to prepare 50% glycerol stocks. Radial growth was determined by point inoculation of GMM plates with a 5-μL drop of a conidial suspension of 2 × 10^6^ conidia/mL, and colony diameters were measured after incubation at 37°C for 96 h. Statistical analysis was performed using one-way analysis of variance (ANOVA) followed by Tukey’s multiple-comparison test with GraphPad Prism 9 software.

Stress analysis was performed through spot dilution (10^4^, 10^3^, 10^2^, or 10 conidia per 5 μL suspension) on GMM agar plates supplemented with H_2_O_2_ (2 mM), FeSO_4_ (0, 30 μM, or 5 mM), calcofluor white (80 μg/mL), Congo red (40 μg/mL), or sorbitol (1.2 M) and incubation at 37°C for 48 h or 72 h.

### Gene-editing strategy and A. fumigatus transformation mediated by CRISPR/Cas9.

In order to correct the *hapE*^P88L^ and *hmg1*^F262del^ mutations in the clinical isolate DI15-105, allele swaps mediated by a Cas9 system were performed, as described previously (26, 53). For reference, all CRISPR RNA (crRNA) sequences and primers (Integrated DNA Technologies) used to build strains are described in Table S1 in the supplemental material, and a detailed scheme of the gene-editing strategy is depicted in Fig. S1 and S2. Repair templates were constructed using WT *hapE* (Afu6g05300) and *hmg1* (Afu2g03700) gene sequences obtained from AF293 genomic DNA. For *hapE* gene manipulation, the WT gene open reading frame (ORF) was cloned into the pAGRP plasmid (54), which contains a phleomycin resistance cassette (PhleoR), using the restriction enzymes NotI and PacI. After confirmation of successful insertion of *hapE* by diagnostic digestion and Sanger sequencing (data not shown), the plasmid (pAGRP-hapE) was used as a template for amplification of the repair template, with primers that incorporated 35-bp microhomology regions with the *hapE* ORF at both the 5′ and 3′ ends.

The DI15-105_ *hapE*^P88L^/*hmg1*^WT-HygR^ strain (26) was created previously, as follows. For *hmg1* gene manipulation, a two-component repair template that consisted of a split *hmg1* allele and a hygromycin resistance marker (HygR) was used. A WT *hmg1* gene sequence, including the ORF and around 50 bp upstream and 500 bp downstream, was PCR amplified using a 3′ primer that inserted 80 bases with homology to the 3′ end of the hygromycin B resistance gene ORF. The HygR cassette was PCR amplified from the pUCGH plasmid (55) using a 3′ primer that introduced approximately 70 bases with homology to the downstream region of *hmg1*.

In order to replace the whole *hmg1* or *hapE* native ORF with a sequence of interest, two separate guide RNAs (gRNA) were selected to generate double-stranded DNA breaks at the 5′ and 3′ regions of the target gene ORF. gRNA duplexes were built using equimolar concentrations of a gene-specific 5′ or 3′ crRNA (see Table S1) and a universal transactivating crRNA (tracrRNA), and Cas9 RNP complexes were then assembled *in vitro* by mixing both 5′ and 3′ gRNA duplexes with the Cas9 enzyme, according to the manufacturer’s instructions. The transformation was carried out by mixing Cas9 RNPs with protoplasts generated from DI15-105 mycelia through enzymatic digestion and 2 to 5 μg of the desired repair template, as described in detail elsewhere (53). Transformation reactions were carried out on sorbitol minimal medium (SMM) agar plates (1.5% agar), and protoplasts were allowed to recover overnight at room temperature. For *hmg1* transformations, SMM top agar (0.75% agar) containing 450 μg/mL hygromycin was added to the plates and incubated at 37°C for 3 to 5 days. For *hapE* transformations, SMM top agar with 375 μg/mL phleomycin was added to the plates and incubated until the next day at room temperature, followed by incubation at 30°C for 3 to 5 days. Single colonies were then subcultured in GMM supplemented with 150 μg/mL hygromycin or 125 μg/mL phleomycin, genomic DNA was extracted, and mutants were confirmed by multiple screening PCRs (see Fig. S1 and S2) and Sanger sequencing (data not shown).

The genetic manipulations performed in this study generated strain DI15-105_*hapE*^WT-PhleoR^/*hmg1*^F262del^, in which the mutated *hapE* allele was replaced with the WT gene sequence, and strain DI15-105_*hapE*^WT-PhleoR^/*hmg1*^WT-HygR^, in which both mutated *hapE* and *hmg1* alleles were replaced with the respective WT gene sequences. The previously constructed DI15-105_*hapE*^P88L^/*hmg1*^WT-HygR^ strain (26) was used as a background to generate the double correction strain DI15-105_*hapE*^WT-PhleoR^/*hmg1*^WT-HygR^.

### Antifungal susceptibility testing.

Antifungal susceptibility to all clinically available mold-active triazoles was determined using gradient diffusion test strips and broth microdilution assays. VRC, ISA, ITRA, and POS strips were applied in the center of RPMI 1640 agar plates that had been inoculated with 500 μL of a suspension of 2 × 10^6^ conidia/mL. The plates were incubated for 48 h at 35°C, and the MIC was defined where a zone of growth inhibition intercepted the strip reading scale. Triazole broth microdilution assays were performed according to CLSI M38-A2 methodology with modifications (49). Briefly, 2-fold serial dilutions of triazole drugs were distributed in U-bottomed 96-well plates, followed by a suspension of A. fumigatus conidia with a final concentration of 2 × 10^4^ conidia/mL. The final drug concentrations tested varied from 0.03125 to 32 μg/mL. The plates were incubated for 48 h at 35°C, and MICs were determined.

### Determination of *cyp51A* expression levels.

The *cyp51A* expression levels of DI15-105 and derived strains were assessed using RT-qPCR. Briefly, RPMI 1640 medium was inoculated with A. fumigatus conidia at a concentration of 2 × 10^4^ conidia/mL and incubated at 35°C and 250 rpm for 24 h or 48 h. Cultures supernatants were discarded, and mycelia were harvested, washed in sterile distilled water, placed in screw-cap tubes, and immediately frozen in liquid N_2_. RNA extraction was performed using the RiboPure-Yeast kit (Invitrogen) according to the manufacturer’s instructions, followed by an extra purification step with isopropanol. RNA concentrations were determined using a NanoDrop spectrophotometer, and 0.5 μg of RNA was used for cDNA synthesis with the RevertAid first-strand cDNA synthesis kit (Thermo Fisher Scientific). qPCR was performed using the SsoAdvanced universal SYBR green supermix (Bio-Rad) with 1 μL of a 1:50 cDNA dilution as the template. The *tubA* gene, which encodes β-tubulin A, was selected as the housekeeping gene for data normalization, and changes in gene expression among isolates were calculated using the 2^−ΔΔ^*^CT^* method with the average Δ*C_T_* of DI15-105 as the control. Primers are listed in Table S1 in the supplemental material. Experiments were performed using four biological replicates, each analyzed in technical duplicates. Statistical analysis was performed using one-way ANOVA followed by Tukey’s multiple-comparison test with GraphPad Prism 9 software.
