# Supplementary material for: hapE and hmg1 Mutations Are Drivers of cyp51A-Independent Pan-Triazole Resistance in an Aspergillus fumigatus Clinical Isolate
Source: Microbiol Spectr. 2023 May 4;11(3):e05188-22. doi: 10.1128/spectrum.05188-22 (PMC10269825; doi:10.1128/spectrum.05188-22)
Supplement: Supplemental file 1 — Supplemental material. Download spectrum.05188-22-s0001.pdf, PDF file, 3.0 MB [file spectrum.05188-22-s0001.pdf]

SUPPLEMENTAL MATERIAL

Table S1. crRNA and primers sequences used in this study.

	Name	Sequence	Description
crRNAs	hapE crRNA 5'	GTTCCATGTCTGAGATTTTC	*hapE* 5' gRNA complex
	hapE crRNA 3'	CGTATTTGAAGCCCGTTGTG	*hapE* 3' gRNA complex
	hmg1 crRNA 5'	ATTTTGTCTATGATAGACAA	*hmg1* 5' gRNA complex
	hmg1 crRNA 3'	CCAACGATTGCCAAAGGTCA	*hmg1* 3' gRNA complex
Primers	P1	TTTTGCGGCCGCATGGAACAGTCTTCGCAGAGC	Cloning of *hapE* in pAGRP
	P2	GGCCTTAATTAATTAGGAAGAGTTTTGGCATACCTGCTGTG	Cloning of *hapE* in pAGRP
	P3	TCTTGTACTTGCCGCTTCTGTTGACTTCGTTGTTGCTAGAAAATCTCAGACATGGAACAGTCTTCG	Repair template for *hapE* gene manipulation
	P4	TTCGAAAGGAGCATGGTTAGCTTAATCGCGTACCGACATGCGGAGAATATGGAGCTTCATCGAATC	Repair template for *hapE* gene manipulation
	P5	TGGGAGACCTCTCGCGATAC	External screening primer to confirm *hapE* gene editing
	P6	CTTTCCAGTATACTCGTTAACCTGCC	External screening primer to confirm *hapE* gene editing
	P7	ATGGCCAAGTTGACCAGTGC	Internal screening primer to confirm *hapE* gene editing
	P8	TCAGTCCTGCTCCTCGGC	Internal screening primer to confirm *hapE* gene editing
	P9	AGCCCGCATAGTGAGTTCTG	Sequencing primer for *hapE*
	P10	CCAATTGCCACTGGCCCG	Sequencing primer for *hapE*
	P11	GACAGGCGGCTTACACCGCCTCTCCTGCTCGCCATTTTGTCTATGATCACTGATGGCTACCTCTCTGATT	Repair template for *hmg1* gene manipulation (*hmg1* allele)
	P12	TCTGGACCGATGGCTGTGTAGAAGTACTCGCCGATAGTGGAAACCGACGCCCCAGCACTCGTCCGAGGGCAAAGGAATAGGCTGCGTTACTCGGTCTTGG	Repair template for *hmg1* gene manipulation (*hmg1* allele)
	P13	CCACTATCGGCGAGTACTTCTACAC	Repair template for *hmg1* gene manipulation (HygR)
	P14	CCCACCTGGGATGAAGCAAAAGCGGGTACATAGATAGGTTTATGGGGACTTTGGGAAGTGAAATATGGGCGAGCTCCCAAATCTGTCCAG	Repair template for *hmg1* gene manipulation (HygR)
	P15	GCCATAGTGTCTCACCCCTG	External screening primer to confirm *hmg1* gene editing
	P16	CGTTCGATGCCACCGAAC	External screening primer to confirm *hmg1* gene editing
	P17	CGAGCTCCCAAATCTGTCC	Internal screening primer to confirm *hmg1* gene editing
	P18	GAGTGCTGCTCGGGGATTG	Internal screening primer to confirm *hmg1* gene editing
	P19	ATATGGCAGCATAACGTAAGGC	Sequencing primer for *hmg1*
	P20	ATCTGCGAACACTGCTGGAG	Sequencing primer for *hmg1*
	P21	GGTCCTCGTTCTCCATGGTC	Sequencing primer for *hmg1*
	P22	CCCTAAGATCGACTTGAACCCC	Sequencing primer for *hmg1*
	P23	CGGTGATCAGTTCACTGCTATGTTCC	*tubA* qPCR
	P24	CTCCTCGCCGTACTCCTCCTCAC	*tubA* qPCR
	P25	CGTGCAGAGAAAAGTATGGCGATATCTTC	*cyp51A* qPCR
	P26	GTCGTCAATGGACTATAGACCTCTTCCG	*cyp51A* qPCR

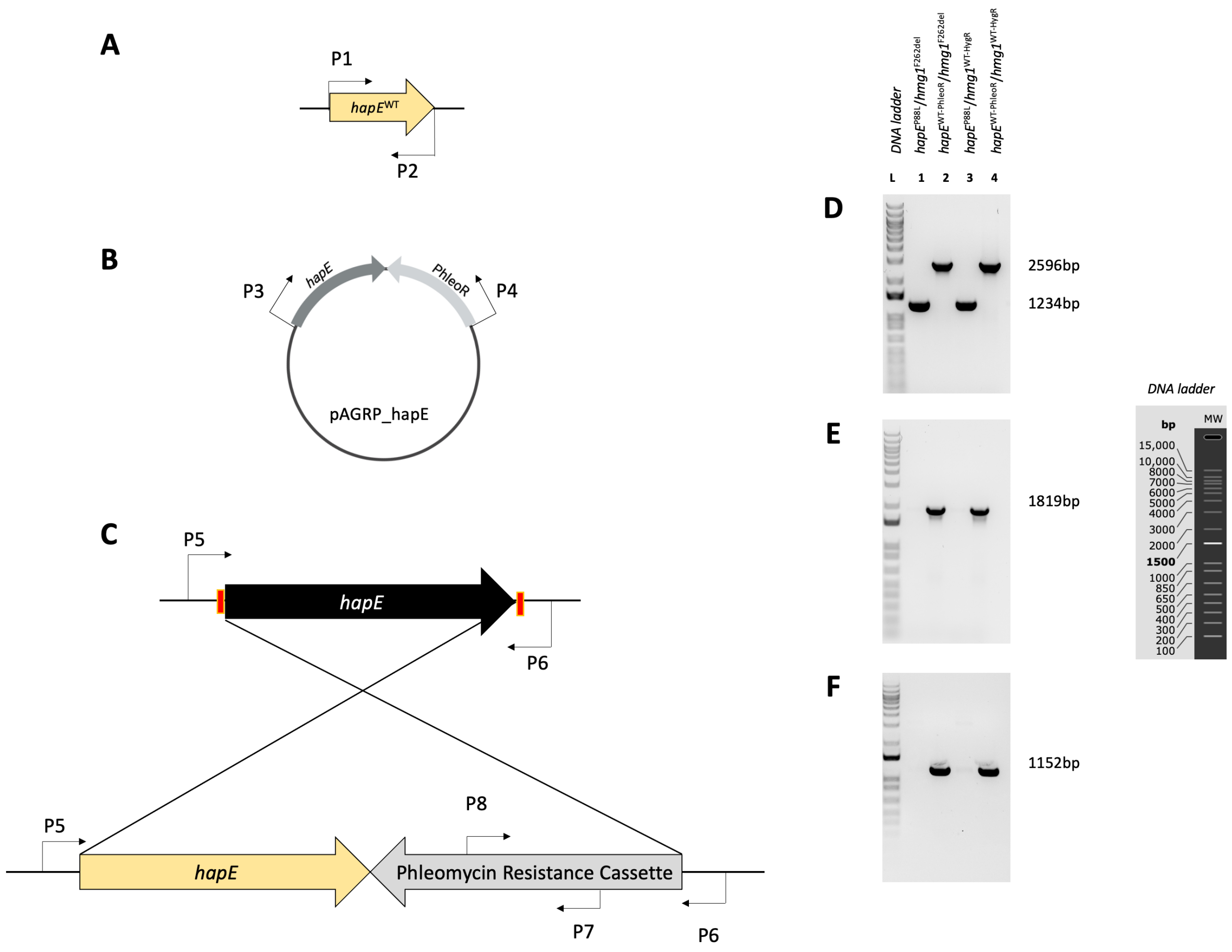

Figure S1. *hapE* gene manipulation strategy. *hapE* gene sequence was amplified from AF293 (*hapE*^{WT}) genomic DNA using primers P1 and P2 (A) and cloned into the pAGRP plasmid containing a phleomycin resistance cassette (PhleoR) using the NotI and PacI restriction sites. A repair template containing the *hapE* gene sequence flanked by PhleoR was amplified from pAGRP_hapE plasmid using primers P3 and P4 (B). Successful transformation was confirmed by PCR using external and internal primers (C). Screening PCR using external primers (P5 and P6) were used to confirm successful integration of hapE/PhleoR cassette in the *hapE* native locus (non-mutants: 1234 bp; hapE/PhleoR+ mutants: 2596 bp) (D). Screening PCR using external and internal primers were used to confirm correct orientation of hapE/PhleoR cassette: amplification with P5 and P7, and P6 and P8 generated a 1819 bp (E) and a 1152 bp (F) amplicon, respectively, while no bands were detected in non-transformants. Red boxes represent PAM sites in *hapE* native locus.

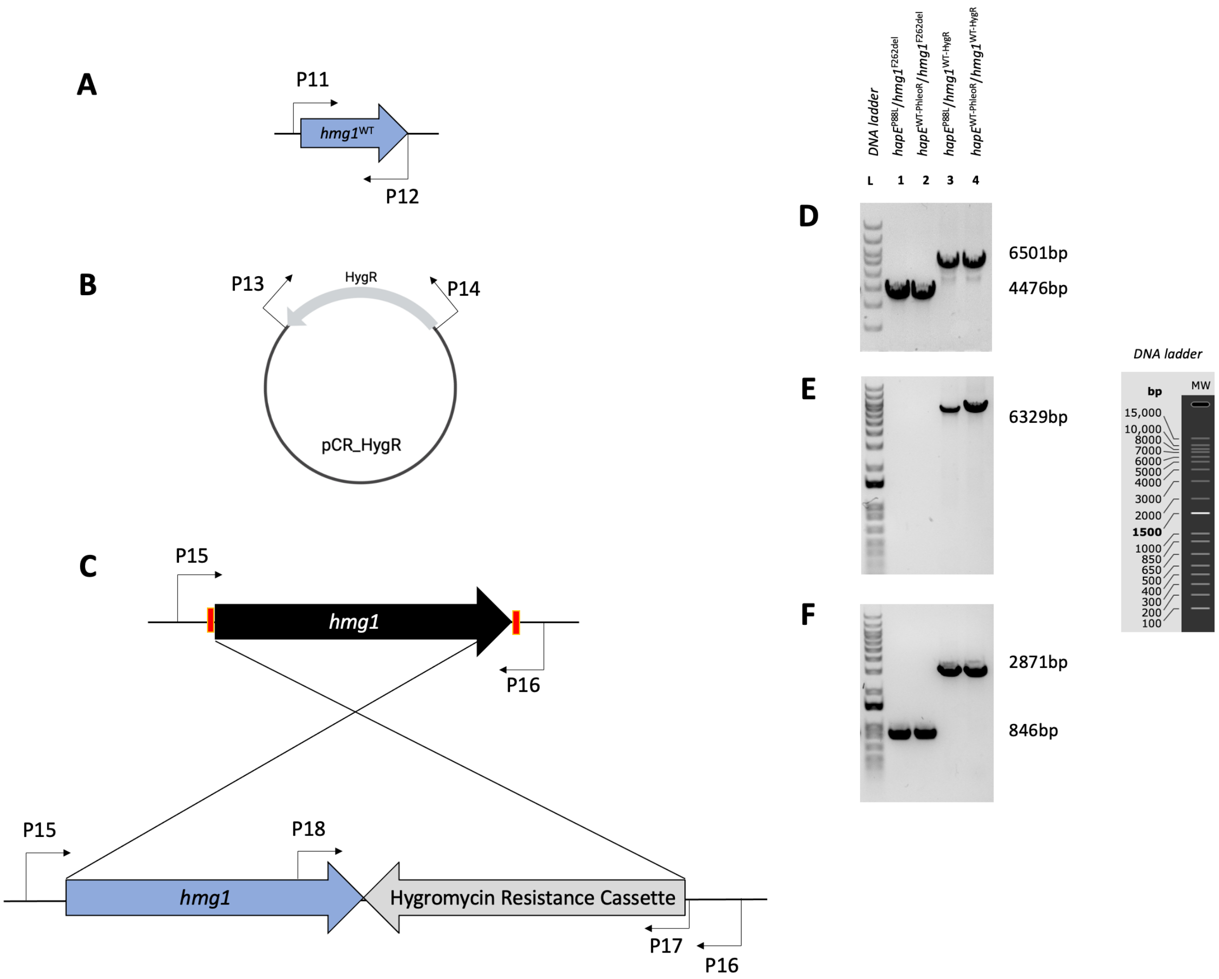

Figure S2. *hmg1* gene manipulation strategy. *hmg1* gene manipulation was performed in a previous study [25] using a two-component repair template that consisted of a split *hmg1* allelle and a hygromycin resistance marker (HygR). The *hmg1* allele was generated by PCR from AF293 (*hmg1*^{WT}) genomic DNA using primers P11 and P12 (A). Primer P12 incorporated 80 bases with homology to the 3′ end of the hygromycin B resistance gene ORF. Meanwhile, the hygromycin resistance cassette (HygR) was amplified from the pCR-HygR plasmid using plasmids P13 and P14 (B). P14 introduced approximately 70 bases of homology with the downstream region of *hmg1*. Both amplicons were used as a repair template in the transformations. Successful transformation was confirmed by PCR (C). Screening PCR using external primers (P15 and P16) were used to confirm successful integration of *hmg1*/HygR cassette in the *hmg1* native locus (non-mutants: 4476 bp; hapE/PhleoR+ mutants: 6501 bp) (D). Screening PCR using external and

internal primers were used to confirm correct orientation of *hmg1*/HygR cassette: amplification with P15 and P17 generated a 6329 bp (E) while no bands were detected in non-transformants; amplification with P16 and P18 generated a 846bp or 2871 bp (F) amplicon for non-transformants and *hmg1*/HygR+ transformants, respectively. Red boxes represent PAM sites in *hmg1* native locus.

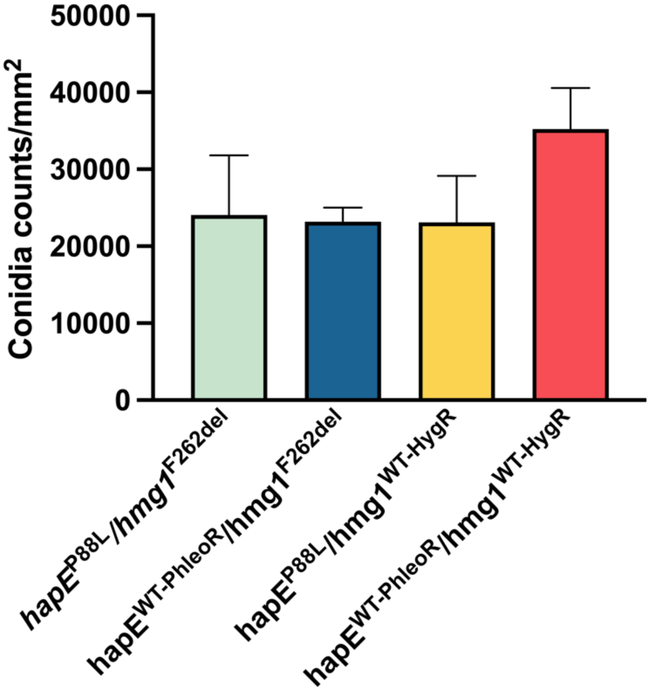

Figure S3. Conidia production of DI15-105 after correction of *hapE*^{P88L} and *hmg1*^{F262del} mutations was assessed by point inoculation of 10^4/5µl *A. fumigatus* conidia in GMM agar and incubation for 96h at 37˚C. Experiment was performed using three biological replicates. Statistical analysis was performed using One-way ANOVA with Tukey's multiple comparisons test.

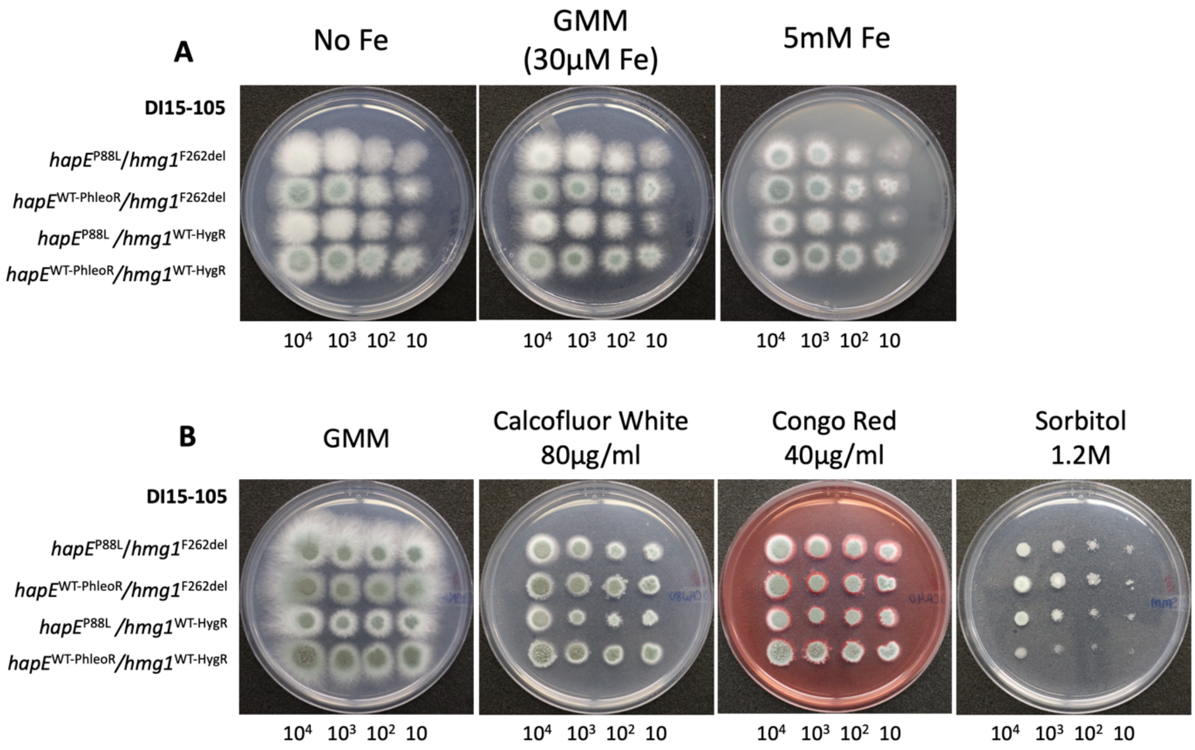

Figure S4. Iron, cell wall and osmotic stress phenotypes of DI15-105 after correction of $\mathit{hapE}^{\mathrm{P88L}}$ and $\mathit{hmg1}^{\mathrm{F262del}}$ mutations. Fungal growth under stress conditions was analyzed through spot dilution assay in GMM agar supplemented as indicated using 5µl suspensions containing 10^4, $10^{3,}$ 10^2 and 10 conidia/5µl followed by incubation at 37˚C for 48h (A) or 72h (B). Experiments were performed using three biological replicates.

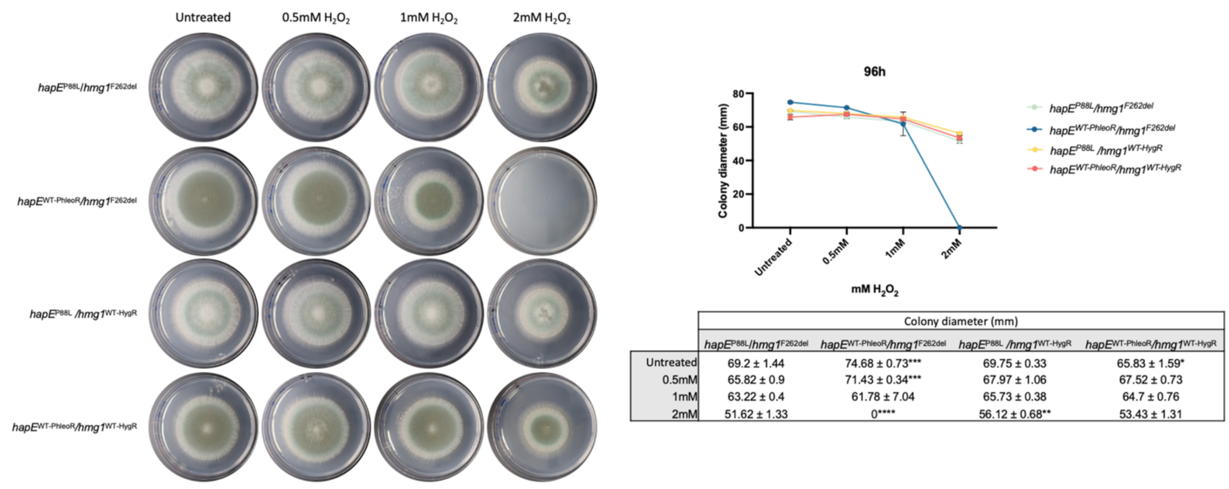

	Colony diameter (mm)			
	$hapE^{P88L}/hmg1^{F262del}$	$hapE^{WT\text{-}PhleoR}/hmg1^{F262del}$	$hapE^{P88L}$ $/hmg1^{WT\text{-}HygR}$	$hapE^{WT\text{-}PhleoR}/hmg1^{WT\text{-}HygR}$
Untreated	69.2 ± 1.44	74.68 ± 0.73***	69.75 ± 0.33	65.83 ± 1.59*
0.5mM	65.82 ± 0.9	71.43 ± 0.34***	67.97 ± 1.06	67.52 ± 0.73
1mM	63.22 ± 0.4	61.78 ± 7.04	65.73 ± 0.38	64.7 ± 0.76
2mM	51.62 ± 1.33	0****	56.12 ± 0.68**	53.43 ± 1.31

Figure S5. Radial growth of DI15-105 after correction of $hapE^{P88L}$ and $hmg1^{F262del}$ mutations in the presence of H_2O_2. Fungal growth was analyzed through colony diameter measurements after 10^4 conidia/5µl were spot inoculated in GMM agar supplemented with 0.5, 1 or 2mM of H_2O_2 followed by incubation at 35˚C for 96h. Experiments were performed independently twice using three biological replicates. Statistical analysis was performed using One-way ANOVA with Tukey's multiple comparisons test, where *p<0.05, **p<0.01, ***p<0.001 and **** p<0.0001 in comparison to DI15-105 ($hapE^{P88L/F262del}$).
